# The effect of foot posture on static balance, ankle and knee proprioception in 18-to-25-year-old female student: a cross-sectional study

**DOI:** 10.1186/s12891-023-06678-2

**Published:** 2023-07-04

**Authors:** Maryam Ghorbani, Rasoul Yaali, Hassan Sadeghi, Tony Luczak

**Affiliations:** 1 Department of Motor Behavior, Faculty of Physical Education and Sport Sciences, Kharazmi University of Tehran, Tehran, Iran; 2 Department of Biomechanics and Sports Injuries, Faculty of Physical Education and Sport Sciences, Kharazmi University of Tehran, Tehran, Iran; 3 NSPARC, Mississippi State University, Mississippi, USA

**Keywords:** Balance control, Position sense, Flexible pes planus, Ankle and knee joints

## Abstract

**Background & purpose:**

Afferent input from the sole affects postural stability. Cutaneous reflexes from the foot are important to posture and gait. Lower-limb afferents alone provide enough information to maintain upright stance and are critical in perceiving postural sway. Altered feedback from propreoceptive receptors alters gait and patterns of muscle activation. The position and posture of the foot and ankle may also play an important role in proprioceptive input.Therefore, the current research aims to compare static balance and ankle and knee proprioception in people with and without flexible flatfeet.

**Methodology:**

91 female students between the ages of 18 and 25 voluntarily participated in this study, of which 24 were in the flexible flatfoot group and 67 were in the regular foot group after evaluating the longitudinal arch of the foot. The position sense of ankle and knee joints were measured using the active reconstruction test of the ankle and knee angle; Static balance was measured using the Sharpened Romberg test. Data were non-normally distributed. Accordingly, non-parametric tests were applied. The Kruskal-Wallis test was applied to compare differences between groups in variables.

**Result:**

Kruskal-Wallis test showed a significant difference between two groups of flat feet and normal feet in the variables of static balance and position sense of ankle plantarflexion, ankle dorsiflexion, and knee flexion (p ≤ 0.05). A significant correlation was found between static balance and sense of ankle and knee position in the group with normal feet. The analysis of the regression line also showed that ankle and knee position sense could predict the static balance score in the regular foot group (ankle dorsiflexion position sense 17% (R^2^ = 0.17), ankle plantarflexion position sense 17% (R^2^ = 0.17) and knee flexion position sense 46% (R^2^ = 0.46) explain of changes in static balance).

**Discussion & conclusion:**

Flexible flatfoot soles can cause loss of balance and sense of joint position; therefore, according to this preliminary study, clinicians must be aware and should take into account this possible deficit in the management of these patients.

## Introduction

The condition of the soles plays a critical role in the quality of daily activities such as standing, walking, and running [1–3]. Any change in the structure of the sole, including increasing or decreasing its arch, is among the factors that expose the foot to overuse injuries caused by physical activity; it is assumed that defects in the quality of coordination, flexibility, and resistance of the foot arch and change in the kinetics of the foot disrupt the function of the foot and make people prone to balance disorders [1, 2, 4]. While almost 80% of people have foot problems in some way, the most common of these disorders at any age is flat foot deformity [5]. According to previous studies, the prevalence of flat foot in the general population was about 25% [6, 7]. This abnormality seems familiar in women [the talar facet of the calcaneus was oriented more medially with respect to the calcaneal body in females than in males [8]], people with higher body mass index (BMI), and people with larger feet [6, 7, 9]. The occurrence of a flat foot in infancy and early childhood is normal, with an arch developing as the individual ages [10].

The occurrence of flat foot deformity can be due to several factors. It may be present from birth (congenital flexible flatfoot), or it may develop in the later stages of life (acquired flatfoot) [11]. Suggested causative factors for acquired flat foot include age, obesity, and not wearing shoes in early childhood [7, 9, 12]. In addition, improper function of the internal and external muscles of the foot at birth or later has contributed to this abnormality [13]. On the other hand, flat foot abnormality is caused by the laxity of the internal longitudinal arch ligament [14]. Ligament laxity allows the foot to drop inward when weight bearing and the heel to deviate to a valgus position. This shape change can change the mechanics of other joints, ligaments, and tendons along with the alignment of the foot. For example, the deltoid ligament is stretched to counteract the rearfoot valgus. Over time, the posterior tibial and peroneal tendon strain becomes more prominent. Abnormal bone structure, ligament laxity, and chronic injury of joint capsule appear with flat foot [15].

Balance is often used to investigate the reactiveness of the lower limb segments and is defined as maintaining the body’s center of gravity within the individual’s base of support. For an individual to maintain balance in an upright posture, the central and peripheral nervous system components continuously communicate to control the body’s alignment and center of gravity within the base of support [16]. Balance control comes from the interaction of the individual with the task and the environment. Environmental constraints such as the type of the base of support, sensory signs, and cognitive demands affect balance control. Individual constraints include the ability to control body position in space resulting from the complex interaction of the musculoskeletal and nervous systems- collectively referred to as the “postural control system.“ Musculoskeletal components include a joint range of motion, spine flexibility, muscle characteristics, and biomechanical relationships between related body parts [17].

When maintaining balance in a standing position, the activity in the postural muscles increases to counter the gravity forces- called postural tone. Sensory inputs from several sensory systems are sent to the central nervous system to create postural tone, one of which is proprioceptive signals. Proprioceptive input includes information about the position sense of muscle and joints and the movement of external receptors located in the skin [18]. Of course, afferent input from the sole has the most significant effect on positional awareness [19, 20]. The activation of external skin receptor inputs in the sole causes a placing reaction that leads to the mechanical stretching of the foot towards the base of the support surface and, as a result, increases the postural tone in the extensor muscles. Somatosensory inputs from the neck are activated by changing the direction of the head and can also affect the distribution of postural tone in the trunk and limbs [21, 22]. The afferents of the visual and vestibular systems also affect the postural tone.

In addition, effective control of posture requires more than the ability to generate and exert force to control body position in space. To know when and how to apply neutralizing forces, the CNS must accurately picture where the body is positioned in space and whether it is stationary or moving. To do this, the CNS must organize information from sensory receptors throughout the body, including visual, somatosensory (joint mechanoreceptors, epidermal external skin receptors, and muscle receptors), and the vestibular system. Each sensory input provides specific information about the position and movement of the body to the CNS [23, 24]. The integration of sensory inputs is essential for postural control.

The somatosensory system provides information about the position and movement of the body by relying on the base of support for the CNS. In addition, somatosensory inputs throughout the body report information about how body parts are related [17]. The importance of somatosensory inputs in static posture control is such that the reduction of afferent input from the lower limb due to vascular ischemia (anesthesia or freezing) increases the movement of the center of pressure (COP) in the base of the support surface during static standing [25, 26]. However, it seems that somatosensory inputs from all body parts contribute to static balance during standing still [27, 28]. Because according to Holm’s point of view, the afferent information sent from the sensory receptors plays an essential role in (1) directly triggering the reflex response, (2) determining the parameters of voluntary programmed responses, and (3) integrating feedback and feedforward mechanisms to maintain balance in static and dynamic states [29].

Since natural postural control occurs automatically without conscious effort, it has traditionally been assumed that little attentional resources are required during balance control. Attentional resources are the information processing resources needed to complete a task. Dual-task interference occurs when simultaneously tasks are performed, resulting in competition for available attentional resources reducing performance on one or more tasks [17]. Dual-task research has shown that there are high attentional demands for postural control. Furthermore, attentional demands are not constant but very different depending on the postural task, the individual’s age, and balance abilities. Also, attentional demands change according to the sensory context; when sensory inputs for postural control are reduced, the attentional demands associated with maintaining stability increase [30–32].Various studies investigated the effect of foot biomechanics on balance. Among others, Cote and his colleagues (2005) studied the effects of increasing and decreasing the arch of the foot on static and dynamic stability; the results showed that the degree of stability in personalized pronated feet is higher than in supinated feet, but these two groups did not have a significant difference with people with normal plantar arches [16]. On the other hand, Khramtsov et al. (2009) evaluated the level of stability in 112 children aged 7 to 10 years with flat and normal arch feet. The results show that children with flat feet have less vertical stability than people with normal arch feet [33]. Abdulwahab and Kachanathu (2015) studied the effects of different degrees of foot posture on static balance in healthy adults. The results showed that increasing the foot posture index (FPI) affects static balance in healthy people [34]. Song et al. (2021) compared the difference in foot pressure, ground reaction force, and balance ability based on foot arch height in young adults; the results showed no significant difference between the peak vertical force in people with flexible flatfoot and regular foot. However, static balance in people with flexible flatfoots was significantly lower than in people with normal feet [35]. Therefore, the results in the field of foot biomechanical effects on balance and stability are contradictory.

On the other hand, fewer studies have focused on the biomechanical effect of the foot sole on the proprioceptive of the joints of the lower limbs. Only research by Yalcin et al. (2012) measured ankle isokinetic strength and proprioception in patients with flat feet. The results indicated that in people with flexible flatfoot, the error scores of passive reproduction of ankle joint position in eversion for the dominant side were significantly higher compared to the control group. There was no significant difference in the strength of the aurator and invertor muscles between people with flat flexible soles and the control group [36]. Therefore, the biomechanical effect of the sole on the balance and proprioceptive of the joints of the lower limbs is unclear.

In other hand, balance control and ankle proprioception are negatively associated with ankle injuries [37, 38]. In 1984 Tropp et al. found that ankle injuries were almost 4 times more prevalent in soccer players with poor balance in comparison to those with normal balance ability [39]. Similarly, Watson found hurdling athletes and Gaelic football players with poor balance had nearly twice as many ankle injuries relative to their counterparts with normal balance [40]. In addition, balance ability was found to be significantly associated with ankle injury risk in both younger male and female basketball players [41]. A systematic review suggested that poorer balance ability is an intrinsic factor associated with increased ankle injury risk [38].

Similar reports of the relationship between ankle proprioception and ankle injury risk are also noted in the literature. A longitudinal study found ankle proprioception could predict ankle injuries in college basketball players [42]. In addition, basketball players with poorer ankle proprioception used an altered pattern of cocontraction of ankle plantarflexors and dorsiflexors, which in turn resulted in greater impact force at the moment of landing associated with higher risk of ankle injury [43]. Ankle proprioception is one of the intrinsic factors associated with ankle injury, as identified by Witchalls et al. in their systematic review [38].

Ankle injuries often lead to disruption of muscles and tendons with associated damage to inherent mechanoreceptors [44–46], which detrimentally alter the quality of proprioceptive information required for balance control. Unrehabilitated, impaired ankle proprioception after ankle injury can subsequently result in long-term deterioration of postural and balance control [47–49]. Gymnasts, dancers, and military sportsmen with poorer ankle proprioception after injury demonstrate worse performance in both static and dynamic postural and balance control tasks [50–52]. These findings suggest that ankle proprioception is closely related to balance control in sport injuries, and balance ability may be significantly affected by impaired ankle proprioception after injuries.

Therefore, based on these findings and taking into account the fact that in flatfoot deformity, changes in the position of the ankle lead to a change in the muscle synergist during activities - so that, nowadays many studies seek to manage this deformity during sports by using the methods of designing sports shoes in order to prevent sports injuries [45, 53, 54], the objectives of this study were: (1) comparison of balance between subjects with and without flexible flat feet; (2) comparison of ankle and knee proprioception between subjects with and without flexible flat feet; and (3) investigating the relationship between ankle and knee proprioception with balance in subjects with and without flexible flat feet. We hypothesized that there would be between the two groups (1) a difference in balance, (2) proprioception of the ankle and knee; there correlation would be detected between proprioception and balance in normal foot group, but not in flatfoot group probably.

## Methodology

### Design and participants

The current study is a cross-sectional and prospective comparative research conducted in compliance with ethical principles. For this purpose, 91 female students between the ages of 18 to 25 voluntarily participated in this study after completing a written consent form, which, after evaluating the internal longitudinal arch of the foot (using the drop navicular test, which is a simple and reliable method for measure the arch of the foot sole [55]): 24 people were in the flexible flatfoot group - Navicular Drop (ND) of ≥ 10 mm was regarded as flexible flat foot, and 67 people were in the normal foot group - Navicular Drop (ND) of 5–9 mm was regarded as normal foot [Sample size was calculated using G∗power 3.1.9.4 (Franz Faul, Kiel, Germany) based on data from a similar study [56]. A required sample size of 23 was determined by calculating an estimated effect size of 0.4, alpha level of 0.05, and power of 0.75. Consequently (statistical test: Repeated measures, within-between interaction), a total of 46 individuals (23 in each group) were required [56]].

Inclusion criteria in research: being in the age range of 18–25 years, not having a history of congenital abnormalities in the lower limbs or legs, not having a systemic condition that affects the position of the lower limbs or legs, not having a history of trauma or pain in any form feet, lower limbs and lumbosacral region at least 12 months before the study.

Exclusion criteria in research: people with structural flatfeet, professional athletes or people who have regular sports activities, volunteers with apparent symptoms of abnormalities in the lower limbs and feet (except flexible flatfeet), history of neurological, rheumatic, metabolic diseases, Psychological disorders, disorders in the vestibular system, history of balance disorders and frequent positional vertigo, severe trunk abnormalities such as severe scoliosis, hyperkyphosis, etc., taking medication that affects balance before the tests, accompanying pathology (surgery in the last three months, a history of sprain, dislocation, semi-dislocation of the ankle and a significant injury that recently involved the joints of the lower limbs) [57].

### Testing protocol

#### Proprioception of the ankle and knee

**Fig. 1:**
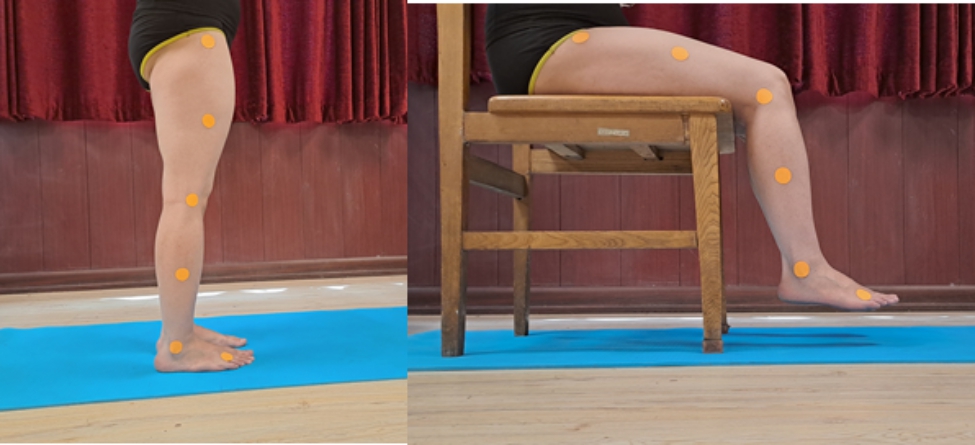
The markers position

In order to measure the position sense of ankle and knee joints, the reconstruction of ankle and knee angles test was used. In this test, the subject sits on a chair so that the angles of the trunk with the thigh and the thigh with the leg are at 90 degrees. The chair’s height was chosen so that the soles of the feet did not reach the ground. In order to eliminate vision, the subject’s eyes were closed with a black blindfold. Then the examiner passively moved the ankle and knee joints subject to the middle range of motion (according to the sources, these angles are 20 degrees for plantarflexion and 10 degrees for dorsiflexion, and 45 degrees for knee flexion [58–60]). Then the subject was asked to actively move her leg and foot up to the intended angle. The subject performed this active angle reconstruction for ankle dorsiflexion, ankle plantarflexion, and knee flexion three times consecutively, then considered the difference between the target angle and the reconstructed angle without considering their sign (absolute error) as the position sense of the ankle and knee joints [61–65]. A goniometer was used to evaluate the person’s performance during the reconstruction of the desired angles (six markers were used to facilitate the measurement, which was the external condyle of the tibia, the outer external malleolus, the distal of the metatarsal bone fourth, the greater trochanter of the femur bone and the midline of the femur bone and the midline of the fibula bone were connected) [61, 62, 65, 66] (Fig. 1).

#### Balance

To evaluate the static balance, the Sharpened-Romberg test was used; for this purpose, the subject stood without shoes on a flat surface and then placed the dominant leg in a tandem position in front of the non-dominant leg; also, the hands were crossed on the chest. This test was performed with eyes closed. The number of seconds the person is able to maintain the position up to 60 s before the balance is disturbed was considered as the person’s score (in case of errors such as: separating the hands from the chest, opening the eyes, shaking, and swaying a lot, the test would stop). Yim-Chiplis et al. (2000) reported the validity of this test with eyes closed as 0.76–0.77 [67].

### Statistical analysis

All information is presented based on mean and standard deviation. Unpaired t-test, Pearson’s correlation coefficient, and linear regression were used for the statistical data analysis. Before performing these tests, the Shapiro-Wilk test was used to check the normality of the data, and the Leven test was used to check the equality of variances. Since in the variables of balance, ankle, and knee proprioception, the normality and equality of variances (homogeneity of variances) were not met; therefore, the equivalent non-parametric test (i.e., Mann–Whitney U) was used. Pearson’s correlation test was first performed to investigate the correlation between balance scores and ankle and knee proprioception variables; then, the linear regression test was performed. Naturally, the normality of the residuals (errors) and the independence of the residuals from each other was investigated. For all calculations, a significance level of 0.05 was considered. All statistical calculations were done using SPSS_24_ software.

## Result

The general characteristics of the subjects by the group are presented in Table 1. As can be seen, in terms of demographic variables between the subjects of the two groups there is significant difference in height, but no difference in weight and Body Mass Index (BMI).

**Table 1:** General characteristics of subjects

Variables	flexible flatfoot group (n = 24)	Normal foot group (n = 67)	T	P value
**age (years)**	19.50 ± 0.72	19.79 ± 1.23	-1.08	0.28
**weight (kg)**	59.92 ± 9.85	57.55 ± 7.29	1.23	0.21
**height (cm)**	163.58 ± 4.79	160.90 ± 4.56	2.44	0.01^*^
**body mass index (kg/m** ^**2**^ **)**	22.37 ± 3.36	22.25 ± 2.86	0.16	0.86

Unpaired t-test to investigate the difference between groups in age, height, and weight variables

P ≤ 0.05: significant difference between groups

Non-parametric Mann–Whitney U test was used to compare the difference between static balance, ankle proprioception (dorsiflexion and plantarflexion), and knee proprioception in the flexible flatfoot group and the normal foot group. The Error of Median of balance score and values of absolute joint positioning error of the flatfoot and control groups are presented in Table 2. The Error of Median of values of absolute joint positioning error in the flatfoot group were higher than those of the control group. whereby a significant difference was noted in the plantarflexion and dorsiflexion position of the dominant ankle and the flexion position of the dominant knee. In other hand Median of balance score in the control group were higher than those of the flatfoot group. As a result a significant difference was noted in the balance score.

**Table 2:** Mann-Whitny U test results

Variables	flexible flatfoot group	Normal foot group	P value	η2
	Median [lower, upper quartile]; Standard Error of Median	Median [lower, upper quartile]; Standard Error of Median		
**Static balance (seconds)**	14.00 [8/00-29.22]; 3.25	36.83 [28.10-45.43]; 1.93	0.00	0.58
**AE of ankle dorsiflexion (degree)**	8.00 [5.00–10.00]; 0.448	8.00 [5.00–8.00]; 0.288	0.03	0.57
**AE of ankle plantarflexion (degree)**	13.00 [10.00–5.00]; 0.670	10.00 [8.00–14/00]; 0.454	0.05	0.57
**AE of knee flexion (degree)**	20.00 [18.00–25.00]; 0.784	18.00 [15.00–20.00]; 0.562	0.01	0.58

P ≤ 0.05: significant difference between groups

AE: Absolute error

η2 = Eta squared

Pearson’s correlation test was used to investigate the correlation between balance test scores and ankle and knee proprioception in the flexible flatfoot group. To investigate this relationship, ankle proprioception (dorsiflexion and plantarflexion) and knee proprioception were considered predictive variables; static balance was considered the criterion variable (Table 3).

**Table 3:** The results of Pearson’s correlation test in the flexible flatfoot group

Variables		AE of Ankle dorsiflexion	AE of Ankle plantarflexion	AE of knee flexion
**static balance (seconds)**	**Correlation Coefficient**	0.274	0.130	0.092
	**P value**	0.19	0.54	0.69
	**N**	24	24	24

P ≥ 0.05: No significant relationship between the variables

AE: Absolute error

According to the data obtained from the correlation coefficient, the values are not significant at the 0.05 level; It can be concluded that ankle and knee proprioception in the group of flatfoot is not correlated with static balance.

Pearson’s correlation test was used to investigate the correlation between balance test scores and ankle and knee proprioception in the normal foot group. To investigate this relationship, ankle proprioception (dorsiflexion and plantarflexion) and knee proprioception were considered predictive variables; the static balance was considered the criterion variable (Table 4).

**Table 4:** The results of Pearson’s correlation test in the group of normal feet and Static balance regression equation based on ankle and knee proprioceptive

Variables		AE of Ankle dorsiflexion	AE of Ankle plantarflexion	AE of knee flexion
**static balance** **(seconds)**	**Correlation Coefficient**	− 0.419	− 0.416	− 0.681
	**t**	10.183	10.219	13.57
	**P value**	0.00	0.00	0.00
	**R**	0.419	0.416	0.681
	**R** ^**2**^	0.176	0.173	0.464
	** F Change**	13.85	13.62	56.21
	**N**	67	67	67

P ≤ 0.05: significant relationship between the variables & the regression equation holds

AE: Absolute error

According to the data obtained from the correlation coefficient, the values ​​are significant at the 0.05 level; it can be concluded that static balance has a relationship with ankle dorsiflexion position sense with a correlation value of -0.309; the static balance has a relationship with ankle plantarflexion with a correlation value of -0.347; the static balance has a relationship with knee flexion position sense with a correlation value of -0.59. Since these coefficients are negative, the relationship between these variables is inverse. This means that balance increases by reducing joint proprioceptive error (increasing proprioceptive).

Therefore, we used the regression test to investigate whether knee and ankle proprioception can predict static balance to investigate the relationship between the variables and the logical connection between the significant correlations (Table 4).

The results showed that ankle and knee proprioception could significantly predict static balance. Respectively, ankle dorsiflexion position sense 17% (R^2^ = 0.17), ankle plantar flexion position sense 17% (R^2^ = 0.17) and knee flexion position sense 46% (R^2^ = 0.46) explains of changes in static balance.

## Discussion

This study aimed to compare static balance and ankle and knee proprioception in people with and without flexible flatfoot. The main result of the present study was that static balance, ankle, and knee proprioception in people with flexible flatfoot were significantly lower than in the group with the normal foot. On the other hand, there was a significant correlation between the scores of the static balance test with ankle and knee proprioception in people with normal feet (that is, ankle and knee proprioception scores can predict static balance scores); however, in the group of flexible flatfoot soles, no significant correlation was found between the scores of the static balance test with ankle and knee proprioception.

The results of the present study were consistent with the studies of Khramtsov et al. (2009), Abdulwahab and Kachanathu (2015), and Song et al. (2021) [33–35]. The results showed that the static balance is significantly lower in the flexible flatfoot group than in the normal foot group (Table 2); in other words, foot biomechanics affects balance and stability in a stationary state. Because the legs with more than 100 muscles, tendons, and ligaments, 26 separate bones, and 33 joints in connection with the ankle, knee, and femur joints form the kinematic chain of the lower limb, regulates the balance of the body in a static and dynamic state. The feet are located at the distal point of this chain and act as a base of support surface for the kinematic chain [68]. Based on this, it is believed that any small dynamic change in the feet affects the control of body position [16]. Also, the structural stability of the foot is maintained by supporting bone structures and soft tissue. Bone supports are created from the contact between the talus bone and the heel bone, while soft tissue support is provided by the deep muscles of the posterior part of the leg and the internal ligaments of the ankle/foot [68].

The posterior tibialis muscle inverts the subtalar joint and locks the bones that form the arch in a stable natural configuration, which is the most common cause of acquired deformity of flatfoot is dysfunction of the posterior tibialis tendon [69]. In addition, the posterior tibialis muscle is the most critical dynamic factor for supporting and maintaining the longitudinal arch of the foot [69]. Changes in the muscular system involved in flexible flatfoot deformity (joint dynamic stabilizers) are believed to affect positional fluctuations (static and dynamic balance) [70]; therefore, this change in the pattern of muscle activity potentially leads to a lack of balance in people with flexible flatfoot. However, the present study’s results were inconsistent with the study of Cote et al. (2005) [16]. A probable cause of dispute in Cote et al.‘s research is due to the use of the Chattecx Balance System to measure positional swing (maximum swing distance recorded in internal-external and anterior-posterior direction) during single-leg standing with eyes open and eyes closed to evaluate balance static; while in this research, the Sharpened-Romberg functional balance test was used (the time a person could maintain the tandem position with eyes closed [67]). Therefore, the use of different methods to evaluate static balance can be the reason for the inconsistency of the two studies.

According to the study of Chiari et al. [71], the significant difference between the static balance between the groups may be attributed to the significant difference in the height subjects - anthropometric factors. But in study of Fabunmi and Gbiri was observed low and positive correlation between Sharpened Romberg Test with foot length [72]. Probably, we cannot attribute the highly significant difference between the two groups in the static balance to the significant difference in the height of the groups.

On the other hand, the research results showed that ankle and knee proprioception are significantly lower in the flexible flatfoot group than in the regular foot group (Table 2). In other words, foot biomechanics affects ankle and knee proprioception. The research results were consistent with the study of Yalcin et al. (2012) [36]. The skeletal structure of the foot and many surrounding soft tissues, such as ligaments, muscles, and tendons, are affected, and their function changes [13, 73]. There are usually mechanical receptors and specialized proprioceptive neurons among these soft tissues. The concept of proprioception is based on the fact that the neurological feedback to the central nervous system (CNS) is inhibited by changing the shape and loading on the soft tissues where the mechanical receptors are located [74]. Therefore, repeated chronic microtrauma of these soft tissues may cause proprioceptive defects [36].

Another consideration is the possible relationship between ligamentous laxity and proprioceptive disorder [75, 76]. It is believed that a flexible flatfoot is associated with different degrees of general ligament laxity [77]. Therefore, ligament laxity may not only lead to flatfoot; it is also one of the causes of lack and defect of proprioception [36]. Also, Lin et al. (2001) point out that people with flexible flatfoots perform physical tasks more poorly compared to people with the regular foot. As determined by gait parameters, they move slowly in the environment [78]. We speculate that these clinical findings of Lin’s study may be related to background proprioceptive deficits.

Also, the research results showed that people with normal foot, ankle, and knee proprioception significantly correlate with balance; in other words, balance scores can be predicted based on ankle and knee proprioception. However, no significant correlation was found between the ankle and knee proprioception and balance in the group of flexible flatfoots. Inputs from mechanoreceptors in the joint capsule, muscle receptors (muscle spindle and Golgi tendon organs), and specialized receptors located in the skin (extrinsic receptors) provide proprioceptive information for static and dynamic postural control [29]. According to Janda, the three critical areas of proprioceptive input for maintaining body position include the foot, the sacroiliac joint, and the cervical spine [29]. In such a way, the afferent input from the sole affects positional awareness [18, 19]. The proprioceptive afferents of the lower limb alone provide enough information for standing straight and are necessary to understand situational fluctuation [29]. Therefore, in people with regular sole foot, the proprioception of the joints of the lower limbs provides the ability to predict balance and positional stability.

On the other hand, according to the study by McKeon and Hertel (2007) and Meyer et al. (2004), in people whose sensory information of the soles of the feet is reduced, visual input is replaced [79, 80]. Likely, in people suffering from the deformity of the flexible flatfoot due to the deficiency that has occurred in the proprioceptive system, the vestibular and vision systems are replacing the lack of sensory information of the sole to control the body position in a static and dynamic state. As a result, in people suffering from flatfoot deformity, proprioceptive scores cannot predict and explain static balance scores.

Also, in these people, postural control probably needs close attention in static and dynamic status. The reduction of somatosensory inputs from the soles of the feet leads to sensory re-weighting by the CNS. On the other hand, the reduction of sensory inputs leads to an increase in attentional demands [30–32]. Since the vision system provides more reliable information between the vision system and the vestibule in such conditions, it seems that the increase in the attention load is more on the vision system. Performing a secondary task does not always have a destructive effect on postural control. Stoffergen et al. (2000) showed that when subjects were asked to focus on a visual target while also performing a visual task (counting the number of letters in a block of text), they fluctuated less than in a single task. The researchers concluded that postural control is organized as part of an integrated perception/action system and can be modified to facilitate the performance of other tasks [81].

In this context, Huxhold et al. (2006) suggested that the improvement in posture control under dual-task conditions may be due to subjects’ attention to a highly automatic process, such as posture control, actually reducing the efficiency of posture control mechanisms, but paying attention to a secondary task may improve the automaticity and increase the efficiency of posture control processes [82]. Other researchers have suggested that reduced volatility in a dual-task environment may be due to increased arousal when performing a secondary task, which improves performance [83]. Therefore, some secondary tasks can increase postural sway (often interpreted as interfering with postural control); still, others reduce postural sway (often interpreted as improving postural control) [17], resulting in the unpredictability of balance based on a sense of proprioception.

Some anthropometric factors of the subjects should be considered in the study to help our understanding of the subject. In the present study, we could not control the height and length of the lower limbs of the subjects (a significant difference was observed between the groups in the height), so the anthropometric characteristics of the subjects should be investigated in future. Also, it is better to examine the proprioception of the joints of the lower limbs, especially the ankle joint, in the frontal movement plane. The relationship between proprioception and dynamic balance should also be investigated.

## Conclusion

The abnormality of the flexible flatfoot can cause loss of balance and sense of joint position; therefore, according to this preliminary study, clinicians must be aware and should take into account this possible deficit in the management of these patients.

## Data Availability

The datasets used and/or analysed during the current study are available from the corresponding author on reasonable request.

## References

[1] Levinger P, Murley GS, Barton CJ, Cotchett MP, McSweeney SR, Menz HB (2010). A comparison of foot kinematics in people with normal-and flat-arched feet using the Oxford Foot Model. Gait Posture.

[2] Williams DS, McClay IS, Hamill J, Buchanan TS (2001). Lower extremity kinematic and kinetic differences in runners with high and low arches. J Appl Biomech.

[3] Tsai L-C, Yu B, Mercer VS, Gross MT (2006). Comparison of different structural foot types for measures of standing postural control. J Orthop Sports Phys Therapy.

[4] Dahle LK, Mueller M, Delitto A, Diamond JE (1991). Visual assessment of foot type and relationship of foot type to lower extremity injury. J Orthop Sports Phys Therapy.

[5] Hogan MT, Staheli LT (2002). Arch height and lower limb pain: an adult civilian study. Foot Ankle Int.

[6] Dare N, Onyije F, Osoma S (2012). Pes planus (flatfoot) in male and female adults of Bayelsa-Nigeria. Electron J Biomed.

[7] Pita-Fernandez S, Gonzalez-Martin C, Alonso-Tajes F, Seoane-Pillado T, Pertega-Diaz S, Perez-Garcia S (2017). Flat foot in a random population and its impact on quality of life and functionality. J Clin Diagn research: JCDR.

[8] Nozaki S, Watanabe K, Kamiya T, Katayose M, Ogihara N (2020). Sex-and age-related morphological variations in the talar articular surfaces of the calcaneus. Annals of Anatomy-Anatomischer Anzeiger.

[9] Pfeiffer M, Kotz R, Ledl T, Hauser G, Sluga M (2006). Prevalence of flat foot in preschool-aged children. Pediatrics.

[10] Bhoir MT. Prevalence of flat foot among 18–25 years old physiotherapy students: cross sectional study.

[11] Shibuya N, Jupiter DC, Ciliberti LJ, VanBuren V, La Fontaine J (2010). Characteristics of adult flatfoot in the United States. J foot ankle Surg.

[12] Sachithanandam V, Joseph B (1995). The influence of footwear on the prevalence of flat foot. A survey of 1846 skeletally mature persons. J bone joint Surg Br volume.

[13] Mulligan EP, Cook PG (2013). Effect of plantar intrinsic muscle training on medial longitudinal arch morphology and dynamic function. Man Therap.

[14] Lobo M, Greisberg J. Adult acquired flatfoot. Foot and ankle: core knowledge in orthopaedics. 2007;1:38–57.

[15] Arachchige SNK, Chander H, Knight A, Flatfeet (2019). Biomechanical implications, assessment and management. The Foot.

[16] Cote KP, Brunet ME, Gansneder BM, Shultz SJ (2005). Effects of pronated and supinated foot postures on static and dynamic postural stability. J Athl Train.

[17] Anne Shumway-Cook MHW. Motor Control: Translating Research into Clinical Practice. 5 ed: LWW; 2017 March, 2016. 640 p.

[18] Grigg P (1994). Peripheral neural mechanisms in proprioception. J Sport Rehabilitation.

[19] Roll R, Kavounoudias A, Roll J-P (2002). Cutaneous afferents from human plantar sole contribute to body posture awareness. NeuroReport.

[20] Kavounoudias A, Roll R, Roll JP (2001). Foot sole and ankle muscle inputs contribute jointly to human erect posture regulation. J Physiol.

[21] Kandel E, Schwartz J, Jessell T. Principles of neural science. 1991.

[22] Roberts TD. Neurophysiology of postural mechanisms. 1978.

[23] Hirschfeld H. On the integration of posture, locomotion and voluntary movement in humans: normal and impaired development. Karolinska institutet; 1992.

[24] Gurfinkel V, Levick YS. Perceptual and automatic aspects of the postural body scheme. 1991.

[25] Asai H. Limiting factor for movable range of the center of foot pressure in backward direction. Vestib Neural Front. 1994:525–8.

[26] Magnusson M, Enbom H, Johansson R, Wiklund J (1990). Significance of pressor input from the human feet in lateral postural control: the effect of hypothermia on galvanically induced body-sway. Acta Otolaryngol.

[27] Andersson G, Magnusson M (2002). Neck vibration causes short-latency electromyographic activation of lower leg muscles in postural reactions of the standing human. Acta Otolaryngol.

[28] Kavounoudias A, Gilhodes J-C, Roll R, Roll J-P (1999). From balance regulation to body orientation: two goals for muscle proprioceptive information processing?. Exp Brain Res.

[29] Izraelski J (2012). Assessment and treatment of muscle imbalance: the Janda approach. J Can Chiropr Assoc.

[30] Lajoie Y, Teasdale N, Bard C, Fleury M (1993). Attentional demands for static and dynamic equilibrium. Exp Brain Res.

[31] Redfern MS, Jennings JR, Martin C, Furman JM (2001). Attention influences sensory integration for postural control in older adults. Gait Posture.

[32] Shumway-Cook A, Woollacott M (2000). Attentional demands and postural control: the effect of sensory context. Journals of Gerontology-Biological Sciences and Medical Sciences.

[33] Khramtsov P, Kurganskiĭ A. Functional stability of the vertical posture in children depending on foot arch condition. Vestn Ross Akad Med Nauk. 2009(5):41–4. 19507353

[34] Al Abdulwahab SS, Kachanathu SJ (2015). The effect of various degrees of foot posture on standing balance in a healthy adult population. Somatosens Motor Res.

[35] Song J-Y, Park S-H, Lee M-M (2021). The comparison of the difference in Foot pressure, ground reaction force, and balance ability according to the Foot Arch Height in Young adults. Annals of Applied Sport Science.

[36] Yalcin E, Kurtaran A, Selcuk B, Onder B, Yildirim MO, Akyuz M (2012). Isokinetic measurements of ankle strength and proprioception in patients with flatfoot. Isokinet Exerc Sci.

[37] Hrysomallis C (2007). Relationship between balance ability, training and sports injury risk. Sports Med.

[38] Witchalls J, Blanch P, Waddington G, Adams R (2012). Intrinsic functional deficits associated with increased risk of ankle injuries: a systematic review with meta-analysis. Br J Sports Med.

[39] Tropp H, Ekstrand J, Gillquist J (1984). Stabilometry in functional instability of the ankle and its value in predicting injury. Med Sci Sports Exerc.

[40] Watson AWS (1999). Ankle sprains in players of the field-games gaelic football and hurling. J Sports Med Phys Fitness.

[41] McGuine TA, Greene JJ, Best T, Leverson G (2000). Balance as a predictor of ankle injuries in high school basketball players. Clin J Sport Med.

[42] Payne KA, Berg K, Latin RW (1997). Ankle injuries and ankle strength, flexibility, and proprioception in college basketball players. J Athl Train.

[43] Fu SN, Hui-Chan CWY (2007). Are there any relationships among ankle proprioception acuity, pre-landing ankle muscle responses, and landing impact in man?. Neurosci Lett.

[44] Peng Y, Wang Y, Wong DW-C, Chen TL-W, Chen SF, Zhang G et al. Different design feature combinations of flatfoot orthosis on plantar fascia strain and plantar pressure: a muscle-driven finite element analysis with taguchi method. Front Bioeng Biotechnol. 2022;10. 10.3389/fbioe.2022.853085 PMC8960448 35360398

[45] Cheng K-W, Peng Y, Chen TL-W, Zhang G, Cheung JC-W, Lam W-K (2021). A three-dimensional printed foot orthosis for flexible flatfoot: an exploratory biomechanical study on arch support reinforcement and undercut. Materials.

[46] Herb CC, Hertel J (2014). Current concepts on the pathophysiology and management of recurrent ankle sprains and chronic ankle instability. Curr Phys Med Rehabilitation Rep.

[47] Munn J, Sullivan SJ, Schneiders AG (2010). Evidence of sensorimotor deficits in functional ankle instability: a systematic review with meta-analysis. J Sci Med Sport.

[48] Yokoyama S, Matsusaka N, Gamada K, Ozaki M, Shindo H (2008). Position-specific deficit of joint position sense in ankles with chronic functional instability. J sports Sci Med.

[49] Nakasa T, Fukuhara K, Adachi N, Ochi M (2008). The deficit of joint position sense in the chronic unstable ankle as measured by inversion angle replication error. Arch Orthop Trauma Surg.

[50] Witchalls JB, Newman P, Waddington G, Adams R, Blanch P (2013). Functional performance deficits associated with ligamentous instability at the ankle. J Sci Med sport.

[51] Witchalls J, Waddington G, Blanch P, Adams R (2012). Ankle instability effects on joint position sense when stepping across the active movement extent discrimination apparatus. J Athl Train.

[52] Forkin DM, Koczur C, Battle R, Newton RA (1996). Evaluation of kinesthetic deficits indicative of balance control in gymnasts with unilateral chronic ankle sprains. J Orthop Sports Phys Therapy.

[53] Song Y, Cen X, Chen H, Sun D, Munivrana G, Bálint K (2023). The influence of running shoe with different carbon-fiber plate designs on internal foot mechanics: a pilot computational analysis. J Biomech.

[54] Song Y, Cen X, Zhang Y, Bíró I, Ji Y, Gu Y (2022). Development and validation of a subject-specific coupled model for foot and sports shoe complex: a pilot computational study. Bioengineering.

[55] Shrader JA, Popovich JM, Gracey GC, Danoff JV (2005). Navicular drop measurement in people with rheumatoid arthritis: interrater and intrarater reliability. Phys Ther.

[56] Kim JS, Lee MY. The effect of short foot exercise using visual feedback on the balance and accuracy of knee joint movement in subjects with flexible flatfoot. Medicine. 2020;99(13). 10.1097/MD.0000000000019260 PMC7220527 32221061

[57] Hedayati R, Fatemi E, Hajihasani A, Ehsani F, Ramezanpour S. The attention needed for balance controlling in young patients with flatfoot. Koomesh. 2016:25–34.

[58] Iris M, Monterde S, Salvador M, Salvat I, Fernández-Ballart J, Judith B (2010). Ankle taping can improve proprioception in healthy volunteers. Foot Ankle Int.

[59] Huston JL, Sandrey MA, Lively MW, Kotsko K (2005). The effects of calf-muscle fatigue on sagittal-plane joint-position sense in the ankle. J Sport Rehabilitation.

[60] Cuğ M, Ak E, Özdemir RA, Korkusuz F, Behm DG (2012). The effect of instability training on knee joint proprioception and core strength. J sports Sci Med.

[61] Tian F, Zhao Y, Li J, Wang W, Wu D, Li Q (2021). Test–retest reliability of a new device Versus a Long-Arm Goniometer to evaluate knee proprioception. J Sport Rehabilitation.

[62] Lephart SM, Pincivero DM, Giraido JL, Fu FH (1997). The role of proprioception in the management and rehabilitation of athletic injuries. Am J Sports Med.

[63] Carpenter JE, Blasier RB, Pellizzon GG (1998). The effects of muscle fatigue on shoulder joint position sense. Am J Sports Med.

[64] Houten D, Cooper D (2017). How does cryotherapy effect ankle proprioception in healthy individuals?. Somatosens Motor Res.

[65] Whitehead PN (2017). Comparing measures of Ankle Proprioception, Strength, and Postural Stability in Male Soccer Players with and without chronic ankle instability as a result of non-contact lateral.

[66] Kaur B, Kaushal K, Kaur S (2019). Effect of cryokinetics on talofibular ligament of improving proprioception of the ankle joint among sports person having ankle sprain. Indian J Physiother Occup Ther.

[67] Yim-Chiplis PK, Talbot LA (2000). Defining and measuring balance in adults. Biol Res Nurs.

[68] Dawe EJC, Davis J, editors. (vi) Anatomy and biomechanics of the foot and ankle. Orthopaedics and Trauma. 2011;25(4):279 – 86.

[69] Imhauser CW, Siegler S, Abidi NA, Frankel DZ (2004). The effect of posterior tibialis tendon dysfunction on the plantar pressure characteristics and the kinematics of the arch and the hindfoot. Clin Biomech Elsevier Ltd.

[70] Kelly LA, Kuitunen S, Racinais S, Cresswell AG (2012). Recruitment of the plantar intrinsic foot muscles with increasing postural demand. Clin Biomech Elsevier Ltd.

[71] Chiari L, Rocchi L, Cappello A (2002). Stabilometric parameters are affected by anthropometry and foot placement. Clin Biomech Elsevier Ltd.

[72] Fabunmi AA, Gbiri C (2008). Relationship between balance performance in the elderly and some anthropometric variables. Afr J Med Med Sci.

[73] Pisal SN, Chotai K, Patil S. Effectiveness of short foot exercises Versus Towel Curl exercises to improve Balance and Foot posture in individuals with flexible flat foot. Indian J Forensic Med Toxicol. 2020;14(3).

[74] Laskowski ER, Newcomer-Aney K, Smith J, Proprioception (2000). Phys Med Rehabil Clin North Am.

[75] Rozzi SL, Lephart SM, Gear WS, Fu FH (1999). Knee joint laxity and neuromuscular characteristics of male and female soccer and basketball players. Am J Sports Med.

[76] Myers JB, Lephart SM. Sensorimotor deficits contributing to glenohumeral instability. Clinical orthopaedics and related research (1976–2007). 2002;400:98–104. 10.1097/00003086-200207000-00013 12072751

[77] Orlin MN, McPoil TG (2000). Plantar pressure assessment. Phys Ther.

[78] Lin C-J, Lai K-A, Kuan T-S, Chou Y-L (2001). Correlating factors and clinical significance of flexible flatfoot in preschool children. J Pediatr Orthop.

[79] Meyer PF, Oddsson LI, De Luca CJ (2004). The role of plantar cutaneous sensation in unperturbed stance. Exp Brain Res.

[80] McKeon PO, Hertel J (2007). Plantar hypoesthesia alters time-to-boundary measures of postural control. Somatosens Motor Res.

[81] Stoffregen TA, Pagulayan RJ, Bardy BtG, Hettinger LJ (2000). Modulating postural control to facilitate visual performance. Hum Mov Sci.

[82] Huxhold O, Li S-C, Schmiedek F, Lindenberger U (2006). Dual-tasking postural control: aging and the effects of cognitive demand in conjunction with focus of attention. Brain Res Bull.

[83] Andersson G, Hagman J, Talianzadeh R, Svedberg A, Larsen HC (2002). Effect of cognitive load on postural control. Brain Res Bull.

